# Effect of Graphene Oxide Nanoparticles on the Physical and Mechanical Properties of Medium Density Fiberboard

**DOI:** 10.3390/polym13111818

**Published:** 2021-05-31

**Authors:** Waheed Gul, Hussein Alrobei

**Affiliations:** 1 Department of Mechanical Engineering, Institute of Space Technology, Islamabad 44000, Pakistan; 2 Department of Mechanical Engineering, Prince Sattam Bin Abdulaziz University, Alkharj 11942, Saudi Arabia

**Keywords:** MDF, graphene oxide, urea–formaldehyde resin, modulus of elasticity, modulus of rupture, internal bond, water absorption, thickness swelling

## Abstract

In this research, the special effects of graphene oxide nanoparticle charging (0, 2, 4, 6, wt.%) on the properties of medium-density fiberboard were examined. Physical and mechanical properties of the panels were determined conferring the method of European Norm standards. The consequences exhibited substantial enhancement in mechanical properties, explicitly in modulus of rupture, modulus of elasticity and internal bonding for 2–6% nanoparticle addition in a urea–formaldehyde resin. The mechanical properties, i.e., internal bond, modulus of elasticity and modulus of rupture were improved by 28.5%, 19.22% and 38.8%, respectively. Results also show a clear enhancement in thickness swelling and water absorption. The physical properties of thickness swelling, water absorption and thermal conductivity were improved up to 50%, 19.5% and 39.79%, respectively. The addition of graphene oxide nanoparticles strongly affected the curing time of the urea–formaldehyde resin and improved its thermal stability.

## 1. Introduction

In the last decade, the properties of graphene materials have been intensively studied in the scientific community owing to their exceptional thermal, mechanical and electrical properties [1]. Graphene is a single layer of carbon atoms arranged in hexagonal rings of an aromatic electron structure. Graphene oxide is the graphene derivatives characterized by the presence of oxygen functional groups on the graphene surface and, therefore, by the presence of defects in the graphene hexagonal rings as well. Such disorder in the graphene structure leads to many unique properties of GO.

Due to the presence of oxygen groups bonded to the surface, GO is an electrical insulator (ideal graphene is an excellent semiconductor). GO is highly hydrophilic and therefore has great wettability. The surface of GO is susceptible to modifications with different molecules, including biological ones, which allows composites with strictly controlled and desired properties to be synthesized. GO, due to its large active surface areas, can serve as a platform for biological molecules (both organic and inorganic) to be safely introduced into the fibers without the risk of uncontrolled and undesirable spreading into the surroundings [2,3].

Composites made of natural fibers have distinguished properties and are the most suitable reinforced materials [4]. Wood-based composite properly called medium-density fiberboard (MDF) is made of urea–formaldehyde (matrix materials) and natural fibers (reinforcement materials) at a ratio of 10:90 wt.% [5]. MDF has been given full attention in recent days, but the major problem with this unique product is its mechanical and physical strength [6]. Many researchers have attempted to improve the mechanical and physical properties of MDF by adding scavengers, additives and reactive agents [7]. Another group of researchers introduced the nanoparticles concept for improving the effectiveness of this composite [8]. Though the researchers had enhanced results in some aspects related to MDF, still there seems to be much room for its improvement. Therefore, the main aim of this research was to conduct experiments on MDF made by the addition of graphene oxide nanoparticles to the urea–formaldehyde resin and analyze its effect on the mechanical and physical properties. The biological performance, i.e., antibacterial activity, can be improved by alginate composite films which are produced with calcium chloride as a cross-linker and several low graphene oxides [9]. Alginate is considered an exceptional biomaterial due to its hydrophilicity, biocompatibility, biodegradability, nontoxicity and low cost in comparison with other biopolymers. Frígols, B. [10] investigated that zinc release, water sorption/diffusion and wettability depended significantly on the type of alginate utilized. Furthermore, Zn^2+^ and GO produced alginate films with increased water diffusion, wettability and opacity.

Surajit, K. et al. [11] examined that binary oxides such as ZnO, TiO_2_, SnO_2_, WO_3_ and CuO when combined with graphene in the form of nanocomposites have excellent potential for detecting trace amounts of hazardous gases and chemicals. In this article, the preparations, characterizations and chemical sensor applications of graphene-oxide nanocomposites were obtained.

Candan, Z. et al. [12] added a combination of ZnO, SiO_2_ and Al_2_O_3_ nanoparticles to a urea–formaldehyde resin and prepared nanofillers. Considering the solid content of urea–formaldehyde, 0.0%, 1.0% and 3.0% of hybrid nanoparticles were added to it. All mechanical properties, i.e., modulus of rupture, internal bonding and modulus of elasticity were admirably improved.

Another special effect of nanoparticles in the form of nano wollastonite was investigated by Taghiyari et al. Nanofillers were produced by adding nano wollastonite in a urea–formaldehyde resin, and camel thorn fibers having a maximum size of one micrometer were mixed with it. Samples of nanocomposites were produced and tested for thickness in swelling and other mechanical properties. A satisfactory improvement was observed in both mechanical and physical properties of nanocomposites [13].

Mechanical and physical properties of nano MDF were also characterized by N. Ismita et al. [14]. In this study, nanoclay was added to the urea–formaldehyde resin at three adsorptions, i.e., 2, 4 and 6%. Mechanical properties, i.e., modulus of rupture and modulus of elasticity were enhanced by 34.1% and 66.0% as compared to conventional composite, respectively, after the addition of 6% of nanofillers. The physical property of thickness swelling was improved by 6.0% only at the same concentration of nanofillers.

Chen et al. [15] introduced methacrylate and calcium carbonate nanoparticles in UF for thermal and mechanical properties of nanocomposites. Mechanical adhesion and bending strength along with thermal weight loss properties were enhanced in a substantial amount.

Da Silva et al. [16] investigated the biological and physical performances of nanocomposites with the help of zinc oxide nanoparticles added in both urea–formaldehyde and melamine–formaldehyde resin. Three concentration levels, i.e., 0.0, 0.5 and 1.0% nanofillers of UF and MF, were mixed with wood fibers, and various samples were prepared from it. The results indicate that optimum biological and physical performance was recorded at 0.5% of MU–zinc oxide nanofillers.

Mechanical, physical and thermal properties of nano MDF were effectively investigated by Alabduljabbar, H. et al. [17]. Alumina nanoparticles were selected as reactive materials with a urea–formaldehyde resin at four different concentration levels, i.e., 0.0, 1.50, 3.0 and 4.50%. At the highest level of nanoparticle concentration, mechanical, physical and thermal properties were improved in a delighted manner.

Research relevant to mechanical impact test on composite structure made of natural fibers is as monitored.

No doubt that many experts of composite materials conducted research to enhance the mechanical and physical strength of the nanocomposite, but at this moment, an optimized result is still pending. It is also a fact that nanotechnology is the latest novel approach to produced nanocomposites with light weight and high strength.

Among all nanomaterials, graphene is the most attractive filler material for manufacturing composite structure because of its comfort amalgamation, extraordinary crop, low cost and adjustability with polymers due to its surface chemistry variations [18].

## 2. Novelty of This Research Work

The main objective of this research work was to develop nano MDF with various quantities, i.e., 0%, 2%, 4% and 6%, of graphene oxide nanoparticles based on the dry content of a urea–formaldehyde resin and to enhance its mechanical and physical performances.

Even though many researchers have worked on nanotechnology, very few researchers have reported on various nanoparticles, i.e., alumina, carbon nanotubes, silicon dioxide, nanoclay and ZnO, and no one used graphene oxide as nanofillers for MDF manufacturing. In the present work, the effect of graphene oxide nanoparticles in a UF resin was studied extensively.

## 3. Materials and Methods

### 3.1. Materials

MDF is typically made up of 82% wood fiber, 10% urea–formaldehyde resin glue and 8% water. Three main raw materials, i.e., urea–formaldehyde resin, graphene oxide (GO) and natural wood fibers were used in this research work.

Urea–formaldehyde resin was purchased from Wah Nobel Company, Taxila, Pakistan. The physical and chemical test result of the resin was provided by the manufacturer, and the desired properties are summarized in Table 1.

Graphene oxide nanoparticles were purchased from Nanjing XFNANO Materials Tech Co., Ltd. (Nanjing, China). The size of the GO nanoparticles ranged from 28 to 40 nanometers. Graphene is defined as a single layer of atom carbon that has a specific characteristic. It is very thin but has spectacular strength. The common process to produce single-sheet graphene is thermal or mechanical treatment of graphene oxide. Due to its unique properties, graphene can be applied in many fields such as energy, environmental and electronic devices application.

Natural (poplar) wood fibers were prepared in a Sunds Defiberator, Sunds Fibertech AB Terminalvägen, Timrå, Sweden machine installed at Ciel Woodworks (PVT) Ltd., Peshawar, Pakistan. The average fiber length was 0.9 mm. Compared to other fiberboards, such as Masonite, MDF is characterized by the next part of the process and how the fibers are processed as individual, but intact, fibers and vessels, manufactured through a dry process. The chips are then compacted into small plugs using a screw feeder, heated for 30–120 s to soften the lignin in the wood, then fed into a defiberator. A typical defiberator consists of two counter-rotating discs with grooves in their faces. Chips are fed into the center and are fed outward between the discs by centrifugal force. The decreasing size of the grooves gradually separates the fibers, aided by the softened lignin between them.

### 3.2. Preparation of UF–GO Nanofillers

The UF–GO nanofillers were synthesized in lab with specific composition summarized in Table 2.

Four samples of nanofillers were prepared by adding 0, 4, 8 and 12 g of graphene oxide to 200 g of a urea–formaldehyde resin. The mixing of the urea–formaldehyde resin and GO nanoparticles was obtained by ultrasonic processor UP 400S of Hielscher Ultrasound Technology Company, USA, for 30 min. Based on the GO nanoparticles’ composition, the samples were assigned named as GOs_1_, GOs_2_, GOs_3_ and GOs_4._

### 3.3. Nano MDF Manufacturing Process

**Figure 1:**
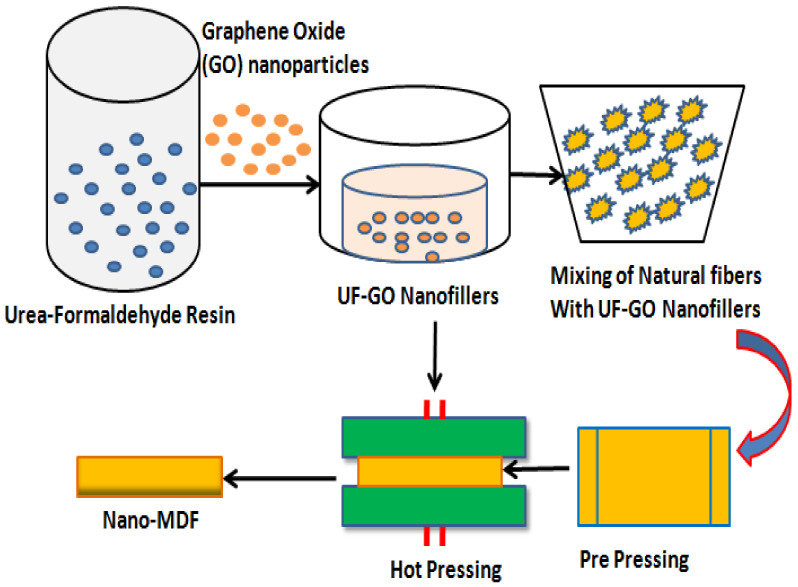
Production process flow of Nano MDF in lab.

The manufacturing process of nano MDF at laboratory scale is shown in Figure 1 as a schematic diagram. Graphene oxide nanoparticles are mixed with urea–formaldehyde resin and stirred with magnetic and ultrasonic sonicator for about 10 and 25 min, respectively. As a result, filler fluids known as UF–GO are formed. These nanofillers are further mixed with natural wood fibers in a mechanically operated drum for about 30 min. At next level, the blended fibers are put in a prepress frame and compressed until 50% reduction in size of the mat occurs. The mat is then pressed in a hot hydraulic press at 170 °C and 165 bar pressure, and finally nano MDF sheet is formed having 16 mm thickness.

### 3.4. Nano MDF Design

First of all, the natural fibers were mixed with UF–GO nanofillers in a rotary drum with a nozzle. Then samples of raw MDF with dimensions 460 × 460 × 17 mm^3^ were manufactured with the help of Berkeley Hydraulic single-opening hot press at 167 bar pressure and 173 °C temperature for 4.1 min. The density of panels was kept in the range of 630–690 kg/m^3^. The raw nano MDF was cooled down for 72 h in a panel treatment section. Finally, the manufactured samples were sanded and trimmed for achieving final size of 430 × 430 × 15 mm^3^.

### 3.5. Characterization

The scanning electron microscopy SEM tests were conducted to evaluate the microstructure of graphene oxide, urea–formaldehyde resin and UF–GO nanofillers. Samples were prepared and passed through Safematic CCU-010 Gold/Carbon Sputter machine, UK. After gold sputtering, SEM was carried out by means of MIRA3 TESCAN Czech Company at maximum 15 KV and magnifications of 50,000× and 70,000× for graphene oxide nanoparticles and 25,000× for cured urea–formaldehyde and UF–GO nanofillers. X-ray diffraction (XRD) of GO nanoparticles was achieved using a GNR X-ray Explorer, Analytical Instrument Group, Novara, Italy, with an output power of 3 kW, output voltage of 60 kV, output current of 60 mA and 2θ range from 5 to 60°. Fourier-transform infrared spectroscopy (FTIR) of UF resin and UF–ZnO nanofillers was carried out using Shimadzu IR Prestige-21Analytical and Measuring Instruments, North America, with a 30° incident angle equipped with a germanium-coated KBr plate, resolution of 16 cm^−1^ and wavenumber range of 500–4000 cm^−1^. Raman spectrum of GO was recorded on a Renishaw 1000 Raman spectrometer with the wavelength of the Raman laser of 532 nm. Energy dispersive spectroscopy (EDS) was also performed with the help of MIRA3 TESCAN Czech Company at maximum 15 KV and magnifications of 25,000× and 50,000×, particularly on the SEM images mapping area. An instrument model QTM500 of Kyoto electronics company, Japan, was carried out to measure thermal conductivity. According to ASTM C 1113–99 standards as reported in [15,16], the tester size was taken 20 × 50 × 100 mm^3^. Three-point bending test and tensile strength test were performed using WDW-30 Electromechanical Universal Testing Machine of JINAN Precision Testing Equipment Company Limited, Jinan, China. Origin 9.0, 32-bit software was used for one-way analysis of variance (ANOVA) with 95.0% confidence level and Tukey Method for the final MDF board.

### 3.6. Mechanical Testing

#### 3.6.1. Three-Point Bending Test

To evaluate the mechanical properties of the medium-density fiberboard, three-point bending test was conducted. The sample size was 370 mm × 50 mm × 15 mm according to EN-310 with loading speed of 4 mm/min. The samples were tested using WDW-30 Electromechanical Universal Testing Machine of JINAN Precision Testing Equipment Company Limited, China.

(1) $$\mathrm{Modulus}\;\mathrm{of}\;\mathrm{Elasticity}=\frac{{\mathrm{PL}}^{3}}{4bd^{3}Y}$$

(2) $$\mathrm{Modulus}\;\mathrm{of}\;\mathrm{Rupture}=\frac{3\mathrm{PL}}{2{\mathrm{bd}}^{2}}$$

#### 3.6.2. Tensile Strength (Internal Bonding) Test

Tensile strength test was performed perpendicular and parallel to the face of the specimen. A 50 mm × 50 mm specimen was glued with a bonding agent to steel or aluminum alloy of similar size. Being a significant characteristic of composite board, internal bond strength was calculated using Equation (3).

(3) $$\mathrm{Internal}\;\mathrm{Bonding}=\frac{P}{\mathrm{bL}}$$

where, P and Y are load and center deflection at proportional limit measured in N and mm, respectively. “b”, “L” and “d” are width, length and depth of the samples measured in mm.

## 4. Results and Discussion

### 4.1. Scanning Electron Microscopy of Graphene Oxide

**Figure 2:**
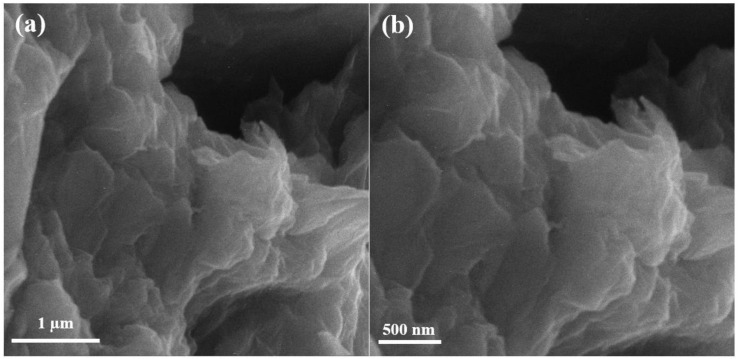
Scanning electron microscopy images of graphene oxide at (**a**) 50,000×, (**b**) 70,000×.

Scanning electron microscopy (SEM) of graphene oxide is shown in Figure 2 below. The SEM results show that graphite peelings have pattern layered configuration. The never-changing graphene oxide discloses arbitrarily combined, shrill wrinkled sheets configuration. It exhibits that the oriented layered structure has been unbalanced due to its oxidation. In Figure 2, the graphite leaves have rubbed each other into monophonic or multi-layer graphene oxide expanses as reported in [17].

### 4.2. X-ray Diffraction of Graphene Oxide Nanoparticles

**Figure 3:**
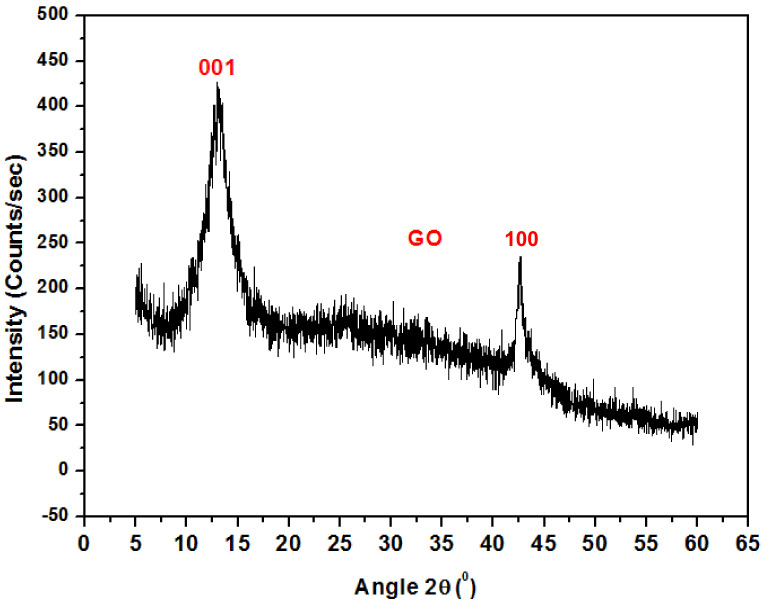
XRD pattern of Graphene Oxide.

Figure 3 shows the XRD pattern for graphene oxide nanoparticles. The spectrum displays a high-pitched peak at 2θ = 12.83° accredited to (001), while the peak at 2θ = 44° is attributed to (100) GO. A similar result was also reported by C. Zhao et al. [18].

### 4.3. FTIR Spectrum of Graphene Oxide

**Figure 4:**
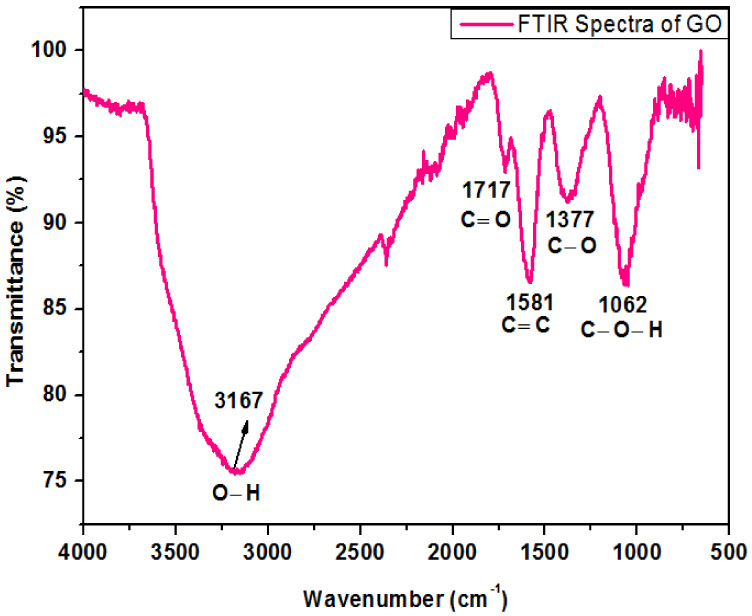
FTIR spectrum of graphene oxide.

Figure 4 shows the FTIR spectrum of oxide nanoparticles. The peak at 3167 cm^−1^ in the high-frequency zone is recognized as an O-H stretching bond and indicates the existence of hydroxyl groups in GO. The band detected at 1717 cm^−1^ was consigned to the carboxyl group. The strident peak found at 1581 cm^−1^ can be assigned to carbon double bond. The peak appearing at 1377 cm^−1^ represents the C–O stretching bond while the peak at 1062 cm^−1^ exhibits vibration mode of C–O–H. These peaks are very close to the previous work reported by Khalili, D. [19].

### 4.4. Raman Spectroscopy of Graphene Oxide

**Figure 5:**
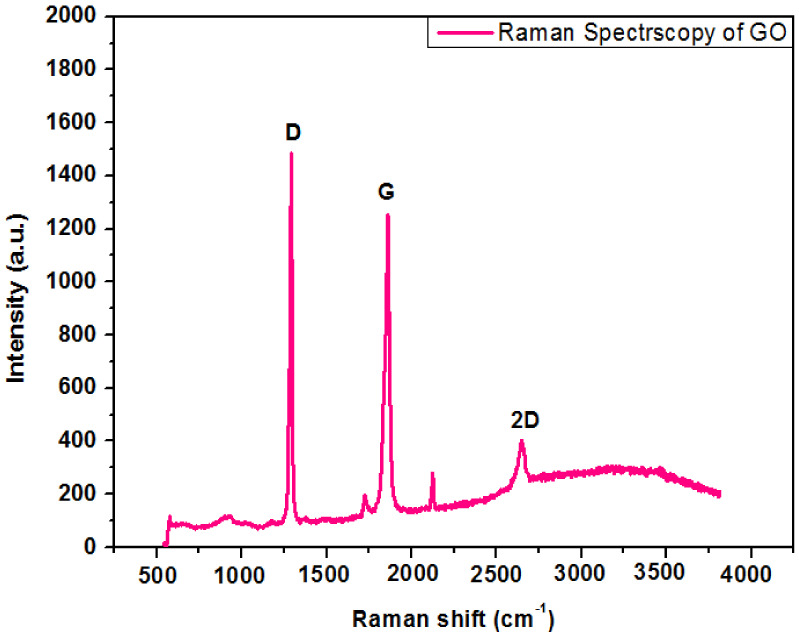
Raman spectroscopy of graphene oxide.

Raman spectroscopy is a fundamental investigation method to govern structural properties of carbonaceous materials. Figure 5 shows the Raman spectrum of GO nanoparticles. Two distinctive peaks occur in the range of 1000–2000 cm^−1^ stating D and G bands in the Raman spectra of GO. The graphene oxide demonstrates a strong with a syndrome tempted peak to peak mode known as G band at 1800–1900 cm^−1^ with a syndrome tempted peak (D band) at 1250–1300 cm^−1^. The G band signifies the appearances of all of the sp^2^ hybrids in a two-dimensional hexagonal linkage. Thus, a perfect crystal should not display the D band. Bahrami, A. et al. [20] reported a comparable reflection of research work.

### 4.5. Scanning Electron Microscopy of UF–GO Nanofillers

**Figure 6:**
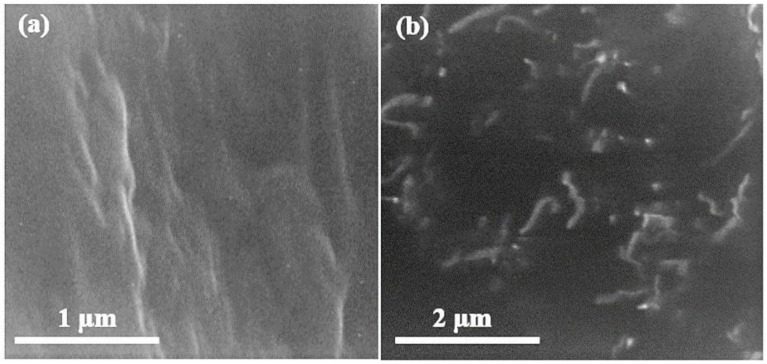
Scanning electron microscopy images of (**a**) cured urea–formaldehyde resin and (**b**) cured UF–GO nanofillers at 25 k.

Figure 6 shows the SEM images of pure UF and UF–GO-cured resin. The cross-linked morphology of the UF resin was not uniform; there were gaps in the cross-linked profile that appear clearly in the SEM image. A peculiar configuration of yokes of the resin was detected, and perceptible minuscule trenches were inspected. These trenches were fenced by 4% GO absorption in the UF resin. The final composite attains high strength due to filling of extra gaps by means of graphene oxide nanoparticles. The observable leaves in the scanning electron microscopy exhibit the presence of GO, and the dark region signifies the urea–formaldehyde resin. The significance was confirmed with energy dispersive spectroscopy (EDS).

### 4.6. Energy Dispersive Spectroscopy (EDS) Analysis

**Figure 7:**
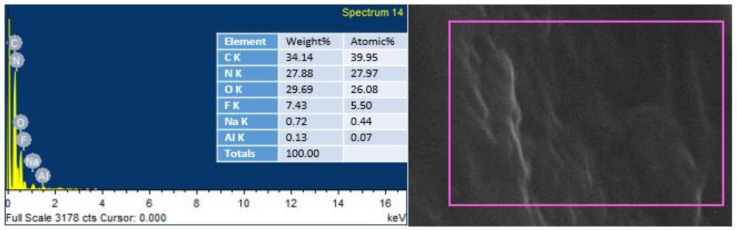
EDS analysis of pure urea–formaldehyde resin.

**Figure 8:**
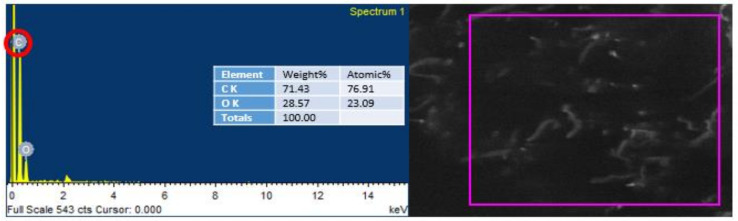
EDS analysis of urea–formaldehyde resin with 4% GO.

EDS was conducted to endorse the existence of GO in the urea–formaldehyde resin observed in SEM analysis. The mapping area for EDS analysis is shown in Figure 7 and Figure 8. One sample with 0% UF–GO resin and another with 4% UF–GO resin were designated for energy dispersive X-ray spectroscopy examination. In the subject analysis, for 4% GO-based resin, the energy peaks of oxygen and carbon at various locations were observed. Energy peaks matched to carbon and oxygen elements confirmed the presence of GO in the UF resin.

### 4.7. Final Physical and Mechanical Characteristics of Nano MDF with ANOVA

**Figure 9:**
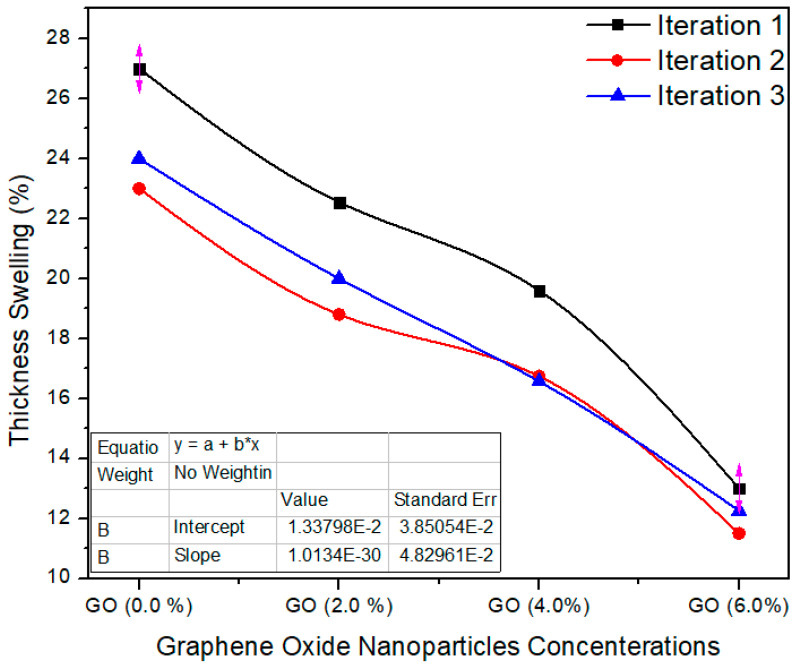
Thickness swelling three iteration values of MDF samples reinforced with graphene oxide nanofillers.

Figure 9 shows the three iteration values of the thickness swelling property. The standard value of thickness swelling is ≤12% as per EN-317 [21]. The thickness swelling average value for the reference sample (containing 0.0% graphene oxide in UF resin) is 24.6%, while for 2%, 4% and 6% nanoparticles concentrations, the mean values of the same property were recorded as 20.45%, 17.63% and 12.25%, respectively. The TS property of the nano MDF reduced with growing nanofiller absorption. The TS property was effectively enhanced by a maximum of 50% after the addition of nanofillers containing 6% of graphene oxide nanoparticles. The average values were considerably altered from each other with a *p* value 0.0003 < 0.05 confidence level. The decrease in TS may be due to nanofillers blocking the pores of the wood fibers and the better curing of the UF resin.

**Figure 10:**
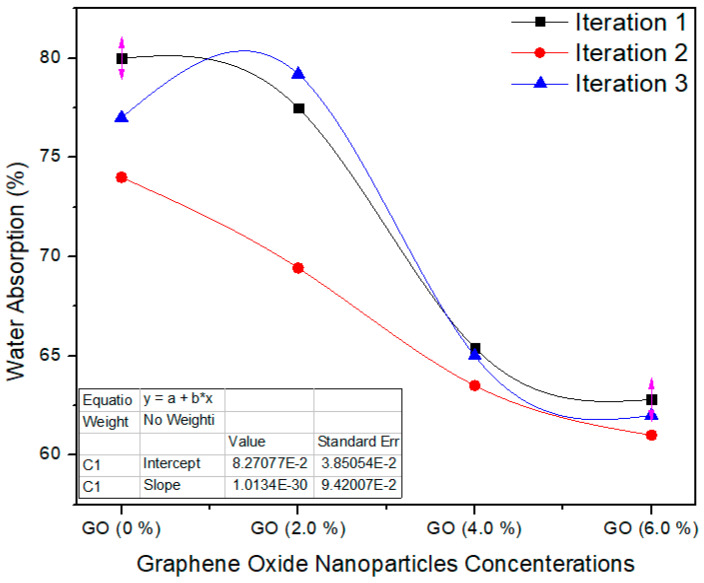
Water absorption property of the final nano MDF with several counts of GO.

Figure 10 shows the water absorption property of the nano MDF boards with all nanofillers filling. The standard value of WA is <45% as per ASTM D570 [22]. The water absorption property diminished with growing nanofiller absorption, and the average values of the tasters were (*p* = 0.0006) superior to the *p* < 0.05 confidence level. The water absorption of the MDF boards was enhanced owing to the improved curing of the boards throughout the hot pressing process. Pores are present in the MDF structure, and therefore MDF absorbed water in bulk amount and WA property becomes weak. By the addition of GO nanoparticles, the pores are covered and produced a superficial layer which reduced the water absorption.

**Figure 11:**
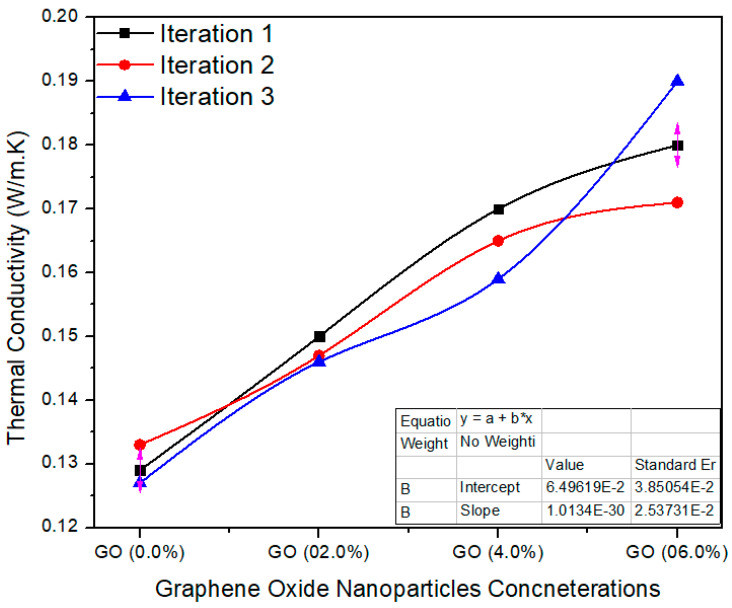
Thermal conductivity of a number of treatments of final MDF with GO.

Figure 11 shows various concentrations of GO in UF for final MDF. The average value of thermal conductivity for 0.0% GO-based nano MDF was noted as 0.129 W/m. K. Meanwhile, 2.0%, 4.0% and 6.0% GO grasping nano MDF panels had average values of 0.147, 0.164 and 0.180 W/m. K. The single factor ANOVA validates the results with probability (*p* = 0.0000290) better than *p* < 0.05 confidence level.

**Figure 12:**
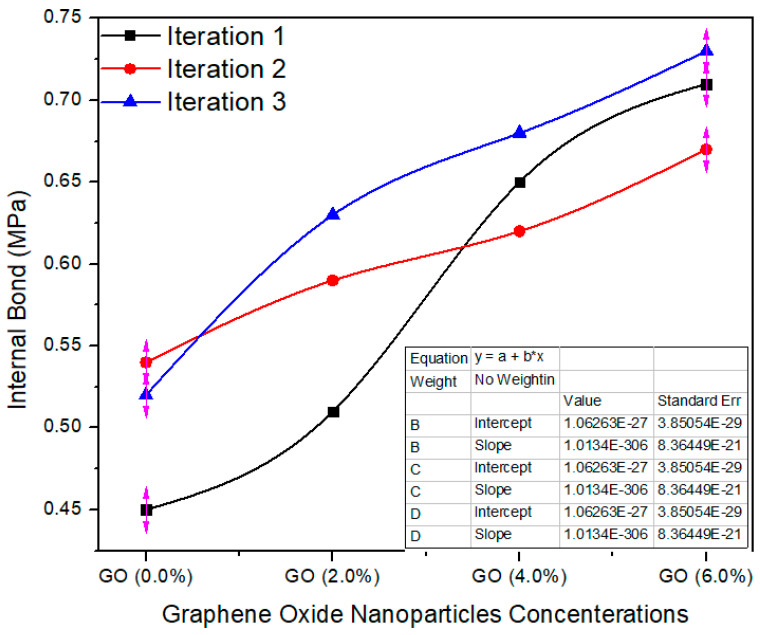
Internal bond values of different counts of GO.

Figure 12 shows the internal bonding property of the MDF board produced with the help of UF and UF–GO resins. The standard value of I.B is 0.7 ± 0.03 MPa as per EN-319 [23]. The average internal bonding value of 0.0% GO-based MDF was 0.50 MPa and 0.57, 0.65 and 0.70 MPa for 2, 4, 6% GO, respectively. The internal bond property of the MDF boards was enhanced meaningfully with aggregate nanofiller absorption. The taster having 6.0% GO had the maximum average value, 0.70 internal bonding. The average of the values of the tasters with the nanofillers was expressively diverse at *p* = 0.0027 less than 0.05 confidence level, as resolute by the ANOVA consequences. The conceivable intention for the improved internal bond of the MDF boards was due to strengthening with graphene oxide nanoparticles upgraded the crosslink compactness of the UF resin; the nanoparticles augmented the degree of heat transfer throughout the hot pressing process of the MDF as reported by [24].

**Figure 13:**
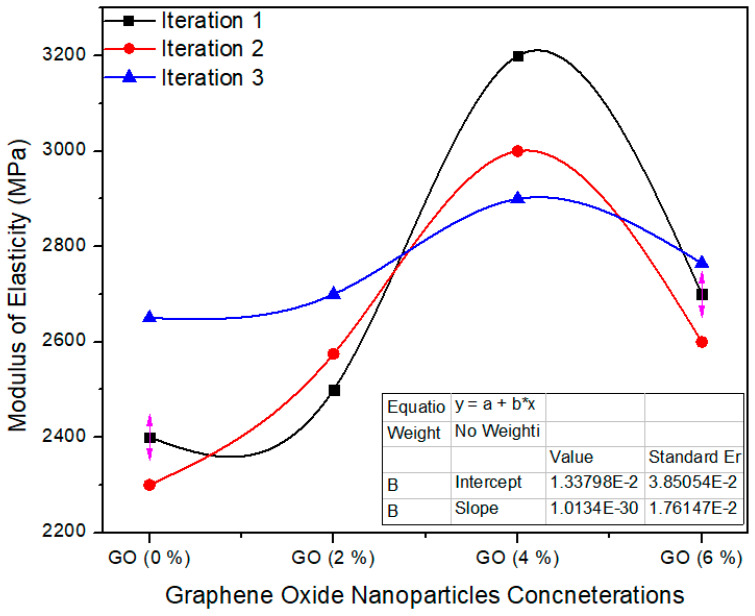
Numerical values of modulus of elasticity (MOE) of various treatments of GO.

Figure 13 shows the one-way ANOVA of MOE for three computations assessment for 0%, 2%, 4% and 6% absorption echelons of graphene oxide nanoparticles. The standard value of MoE is ≥2800 MPa as per EN-310 [24]. A 0.0% GO containing MDF has an average value of 2450 MPa, while 2.0%, 4.0% and 6.0% GO-based MDF has 2591.6, 3033 and 2688 modulus of elasticity average values, respectively. One-way ANOVA extents approve that the probability (*p*-value) is 0.0042. The reduction of MoE value was observed when the highest concentration (6%) of GO was applied to nano MDF. Due to high thermal conductivity value, early and fast curing of the resin occurs which tends to increase brittleness in MDF, which is why we have to use optimum GO nanoparticles for enhancement of MoE.

**Figure 14:**
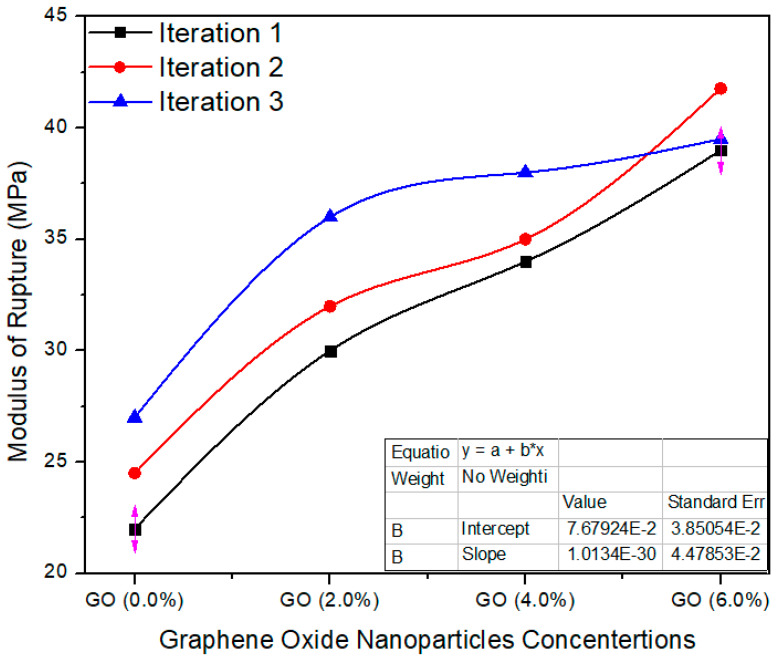
Modulus of rupture vs. % values of counts of GO.

Figure 14 shows the average value assessment between the reference and UF–GO-based tasters at 2%, 4% and 6% graphene oxide concentration levels. The standard value of MoR is ≥25 MPa as per EN-310. The reference tester had an average value of 24.5 MPa; 2%, 4% and 6% Go-based MDF had average values of 32.6, 35.6 and 40.09, respectively. These average values are altered from each other, and the probability (*p* = 0.000257) is better than *p* < 0.05 confidence level. The enhanced MOR of the panels after reinforcement with nanofillers may be due to better curing of the resin during the pressing and to the crosslink density of the UF resin.

## 5. Conclusions

In this research study, nano MDF panels of 16 mm thickness were manufactured with four concentrations levels, 0%, 2%, 4% and 6%, of graphene oxide nanoparticles. From experimental results, the following conclusion may be drawn. The physical and mechanical properties of final MDF were investigated with enough enhancements.

The physical properties thickness swelling, water absorption and thermal conductivity were improved up to 50%, 19.5% and 39.79%, respectively. The decrease in TS may be due to nanofillers blocking the pores of the wood fibers and the better curing of the UF resin. Moreover, pores are present in the MDF structure, and therefore MDF absorbed water in bulk amount and WA property became weak. By the addition of GO nanoparticles, the pores were covered and produced a superficial layer which reduced the water absorption.

On the contrary, the mechanical properties, i.e., internal bond, modulus of elasticity and modulus of rupture were improved by 28.5%, 19.22% and 38.8%, respectively. The conceivable intention for the improved internal bond of the MDF boards was due to strengthening with graphene oxide nanoparticles which upgraded the crosslink compactness of the UF resin; the nanoparticles augmented the degree of heat transfer throughout the hot pressing process of the MDF. Similarly, due to high thermal conductivity value, early and fast curing of the resin occurs which tends to increase brittleness in MDF, which is why we have to use optimum GO nanoparticles for enhancement of MoE. Meanwhile, the enhanced MOR of the panels after reinforcement with nanofillers may be due to better curing of the resin during the pressing and to the crosslink density of the UF resin. Future research can be made to study the biological performances of MDF by the addition of various nanoparticles.

## Figures and Tables

**Table 1:** Chemical and physical properties of Urea–formaldehyde Resin.

Viscosity (Cps)	Density (kg/m^3^)	pH	Free Formaldehyde (mg/100 g)	Gel Time (s)	Solid Content (%)
195–280	1.245	7.8	0.73	62	61

**Table 2:** Composition of UF–GO nanofillers.

Composition				
Materials	GOs_1_	GOs_2_	GOs_3_	GOs_4_
UF (g)	200	200	200	200
GO (g)	0	4	8	12

## Data Availability

The data presented in this study are available on request from the corresponding author.
